# Generation of nonlinearity in the electrical response of yeast suspensions

**DOI:** 10.1038/s41598-022-07308-y

**Published:** 2022-03-04

**Authors:** K. Tamura, M. Muraji, K. Tanaka, T. Shirafuji

**Affiliations:** Graduate School of Engineering, Osaka City University, 3-3-138, Sugimoto, Sumiyoshi-ku, Osaka, 558-8585 Japan

**Keywords:** Biophysics, Cell biology, Microbiology, Engineering

## Abstract

The mechanism through which nonlinearity is generated in the response waveform of the electric current obtained by applying alternating current voltage to yeast suspension has not yet been elucidated. In this paper, we showed that the response waveform depends on the applied voltage and frequency. The results showed that distortion (nonlinearity) in the waveform increases as the applied voltage increases and/or the frequency decreases. We suggest a model for the generation of nonlinearity based on the influx of potassium ions into the cell via potassium ion channels and transporters in the membrane due to the applied voltage. Furthermore, we validated this model by simulating an electrical circuit.

## Introduction

Cells within living organisms transmit information through electrical signals and chemical reactions^1,2^. Characterizing the electrical properties of cells is essential to understanding the mechanisms underlying biological activities.

Yeast is a eukaryote, surrounded by an insulating membrane comprised of a lipid bilayer, which maintains a difference in the concentration of ions between the environments inside and outside of the cell^1,2^. The concentration of ions is controlled by membrane proteins that function as ion channels, transporters and pumps. Their activities generate a voltage difference, known as the membrane potential, between the inside and outside of the cell. Ketchum et al.^3^ reported that potassium ion channels are present in the membrane. Current passing through these channels depends on the membrane potential^3,4^.

A method for electrically measuring the state of cells based on their membrane properties has been demonstrated^5–12^. Kell et al. found that the response waveform showed a unique pattern when sinusoidal alternating current (AC) voltage with low frequency was applied to a suspension of living yeast^5–7^. They suggested that the response waveform included substantial harmonics of the applied voltage, driven mainly by an enzyme (H^+^-ATPase) present in the membrane. Nawarathna et al. observed the generation of harmonics from living yeast and argued that it was caused by an enzyme present in the membrane^13–15^. A nonlinearity generated near the electrode has been reported^10,12,16^. The generation of the nonlinearity depends on the shape of the electrode and is affected by the intensity and distortion of the electric field near the electrode^10^. Although unique factors affecting the generation of harmonics in living yeast, such as the dependence of the generation process on the concentration of yeast in the suspension and the condition of the culture, have been reported, the mechanism underlying this process has not yet been fully elucidated^8–12^.

We report for the first time the response waveform of a current passing through a yeast suspension and the dependence of its nonlinearity (the distortion of waveforms away from a sinusoidal waveform, which is not proportional to the amplitude of the applied voltage) on the applied sinusoidal AC voltage and frequency. Furthermore, to elucidate the mechanism underlying the generation of nonlinearity, we suggest a model based on the influx of potassium ions into the cell via potassium ion channels and transporters in the membrane. We validated this model by simulating an electrical circuit using an equivalent circuit.

## Materials and methods

The yeast used in this paper is *Saccharomyces cerevisiae* BY4741^8–12^. The method of yeast culture has been described previously^11^. We pre-cultured the yeast in YEPG medium (pH 5.0) containing 1% yeast extract, 2% polypeptone, and 2% glucose under anaerobic conditions at 25 °C for 48 h. Then, 4 mL of pre-cultured medium was added to 100 mL fresh YEPG medium and cultured for 24 h. This process leads to yeast in the stationary phase. Current passing through the living and dead yeast suspensions was measured. The method of applying a voltage and acquiring data, together with a flowchart, is provided elsewhere^10,11^. The dead yeast suspension was prepared through 70 °C heat treatment for 3 min.

We applied sinusoidal voltage between the electrodes of the measurement cell containing the yeast suspension and measured the waveform of the current passing through the suspension using an ammeter (Zero Shunt Ammeter HM-103; Hokuto Denko). The size of the measurement cell has been shown previously^11^, and two gold electrodes are set in the measurement cell. The diameter and height of the cylindrical electrodes are 1.0 mm and 6.5 mm respectively and the distance between electrodes is set to 10 mm. In the experiment to assess dependence on the applied voltage, we used a constant frequency of 14 Hz and set the amplitude of the applied voltage to 0.8, 0.9, 1.0, 1.1, or 1.2 V. In the experiment to assess frequency dependence, we used a constant amplitude of 1.2 V for the applied voltage and frequencies of 8, 11, 14, 22, 30, 50, and 100 Hz. The applied voltage was controlled by a computer, converted to an analogue waveform, smoothed with a low-pass filter and applied between the electrodes of the measurement cell. The current measured by the ammeter was amplified with an operational amplifier, passed through an anti-aliasing filter, converted to a digital waveform and sent to the computer. The sampling frequency was set to 2^7^ = 128 times higher than the applied voltage frequency: 1,024 Hz at an applied voltage of 8 Hz, 1,408 Hz at 11 Hz, 1,792 Hz at 14 Hz, 2,816 Hz at 22 Hz, 3,840 Hz at 30 Hz, 6,400 Hz at 50 Hz, and 12,800 Hz at 100 Hz. The electrodes were brushed manually with aluminum oxide prior to each measurement. The temperature of each experiment was maintained at 25 °C.

## Results

**Figure 1:**
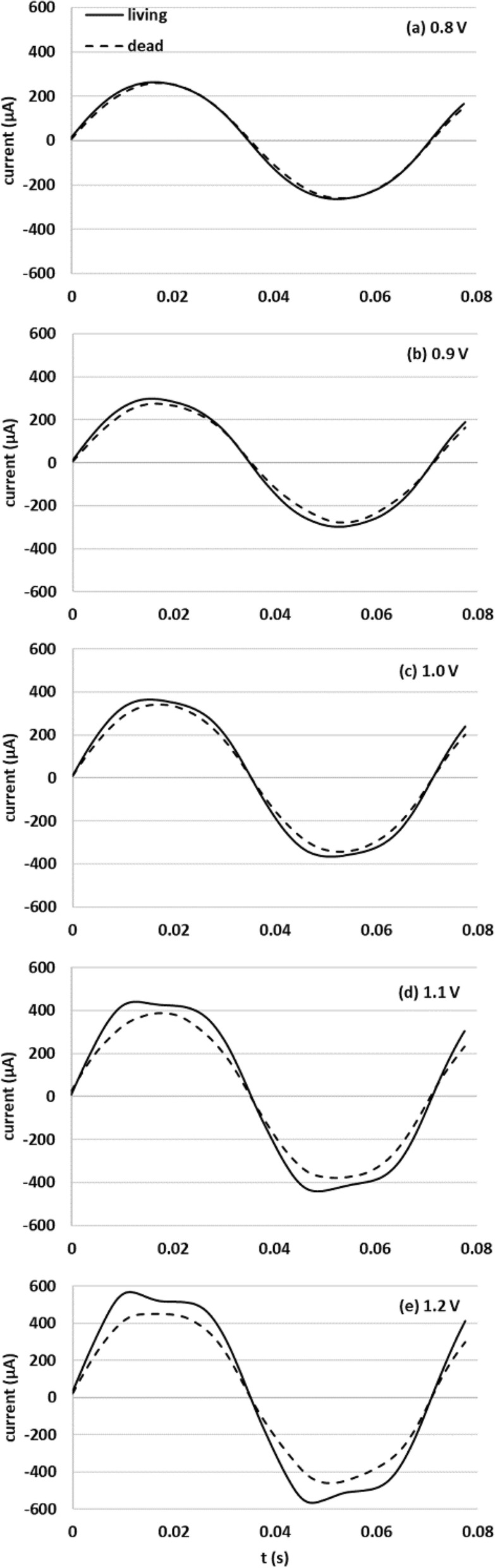
(**a**)–(**e**) Current waveforms of living and dead yeast 90 s after measurement was initiated at applied voltages of 0.8, 0.9, 1.0, 1.1, and 1.2 V at 14 Hz.

Figure 1a–e shows the current waveform in response to voltages of 0.8, 0.9, 1.0, 1.1, and 1.2 V, applied to living and dead yeast suspensions at 14 Hz. Each is the waveform approximately 90 s after measurement began, indicated on the horizontal axis of Fig. 1 as t = 0. The figure shows that the current differed between living and dead yeast, with the depression at the top and bottom of the waveform for living yeast and the separation from a sine wave (distortion) both increasing as the applied voltage increased.

**Figure 2:**
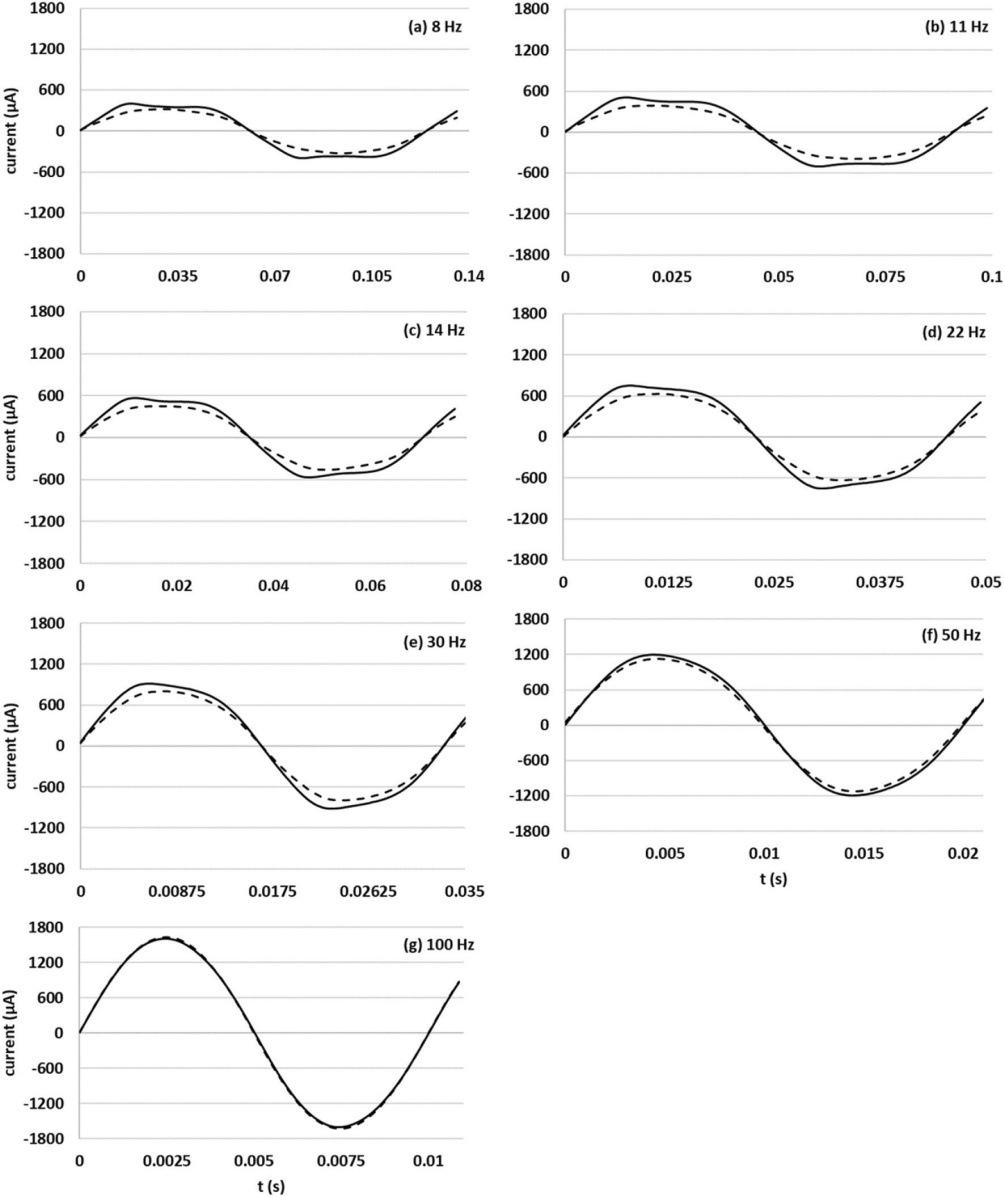
(**a**)–(**g**) Current waveforms of living and dead yeast 90 s after measurement was initiated at frequencies of 8, 11, 14, 22, 30, 50, and 100 Hz at 1.2 V.

Figure 2a–g shows the current waveforms obtained at 8, 11, 14, 22, 30, 50, and 100 Hz with an applied voltage of 1.2 V. Figure 2 shows that the distortion in the waveform of the living yeast decreased and the waveform became similar to that of dead yeast as frequency increased.

We derived the harmonic component of the response waveform by performing fast Fourier transform (FFT) for 1024 samples (eight cycles) approximately 90 s after measurement began after using the Hanning window. We calculated the content of the third harmonic according to the fundamental frequency as an index of nonlinearity^9–11^. Table 1 shows the third harmonic content at each applied voltage at a set frequency of 14 Hz. A significant difference was found between living and dead yeast suspensions at each applied voltage. Table 2 shows the third harmonic content at each frequency with an applied voltage of 1.2 V. A significant difference was found between living and dead yeast suspensions at each frequency. We found that the third harmonic content of the response waveform can be used to distinguish between living and dead yeast.

**Table 1:** Results of the t-test of the third harmonic contents of living and dead yeast suspensions at each voltage tested.

Voltage (V)	Yeast cell	Number of experiments	3rd harmonic content rate average (%)	3rd harmonic content rate standard deviation (%)	Significance level (t-test)
0.8	Living	5	4.1	0.3	P < 0.01
	Dead	4	2.2	0.2	
0.9	Living	5	5.1	0.5	P < 0.01
	Dead	4	2.8	0.2	
1.0	Living	5	7.8	0.3	P < 0.01
	Dead	4	3.8	0	
1.1	Living	5	10.7	0.2	P < 0.01
	Dead	4	5.1	0.5	
1.2	Living	5	12.3	0.2	P < 0.01
	Dead	4	6.2	0.3

**Table 2:** Results of the t-test of the third harmonic contents of living and dead yeast suspensions at each frequency tested.

Frequency (Hz)	Yeast cell	Number of experiments	3rd harmonic content rate average (%)	3rd harmonic content rate standard deviation (%)	Significance level (t-test)
8	Living	5	14.9	0.2	P < 0.01
	Dead	4	6.2	0.7	
11	Living	5	14.1	0.3	P < 0.01
	Dead	4	6.7	0.2	
14	Living	5	12.3	0.2	P < 0.01
	Dead	4	6.2	0.3	
22	Living	5	10.1	0.1	P < 0.01
	Dead	4	5.6	0.3	
30	Living	5	8.0	0.2	P < 0.01
	Dead	4	5.0	0.1	
50	Living	5	4.6	0.1	P < 0.01
	Dead	4	3.1	0.1	
100	Living	5	1.4	0.2	0.01 < P < 0.05
	Dead	4	1.1	0	

## Discussion

As shown in Fig. 1, unique distortion patterns were apparent in the positive and negative peaks of the current waveform obtained from living yeast as the applied voltage increased. Furthermore, the same tendency was found at low frequencies, as illustrated in Fig. 2. As shown in Tables 1 and 2, the third harmonic content increased as applied voltage increased and frequency decreased, and the unique distortion in the current waveform corresponded to that content.

We consider the voltage generated between the inside and outside of the cell under the electric field to have derived from the applied voltage. Assuming that the yeast cell is spherical, the thickness of the membrane is much smaller than the radius of the cell, and the suspension has much greater conductivity than the membrane, the voltage *ΔV* [V] between the membrane generated by the outer electric field* E* [V/m] was given by *ΔV* = *1.5 a E cosθ* ^17^. Here, *a* represents the radius of the cell [m], and *θ* represents the angle between the line connecting the target spot on the membrane and the center of the cell and direction of the electric field. Given that the experimental conditions in this paper meet those assumptions, *ΔV* is approximately 0.7 mV. As this voltage is low, it is unlikely to affect the enzymes present in the membrane^5^.

The interface between the electrode and suspension near the electrode may generate a large voltage drop^16^, but because the thickness of the interface around the electrode is about 2 nm (obtained by solving Poisson’s equation, assuming that the distribution of ions in the suspension follows the Boltzmann distribution and direct current voltage is applied) and the yeast has a cell wall thickness of about 100 nm, the membrane is not included in the interface area^18^. Therefore, the outer electric field cannot affect the enzymes present in the membrane solely based on the intensity of the electric field at the interface.

Previous works^10,12,16^ have considered the site where the nonlinearity is generated. In previous research^10^, the extent of nonlinearity relative to the fundamental wave is higher when cylindrical electrodes are used than plate electrodes, despite their small area. Moreover, the generation of the nonlinearity is closely related to the intensity of the electric field and shows differences depending on the gradient of the electric field near the electrode.

We consider a model related to the spatial pattern of the electrical field near the electrode. In this model, ions in the suspension are first moved by the electrical field, and voltage is then generated by the difference in the amount of ions moved between the inside and outside surface of the membrane when the intensity of the electric field between them differs, affecting the inflow of ions into the cell via channels and transporters present in the membrane. Here, for the inside and outside surface of the membrane, we consider the amount of ions passing through a unit area of the membrane. Assuming that the intensity of the electric field *E*_*i*_ [V/m] is proportional to the applied voltage *V* [V], *E*_*i*_ is expressed with the proportional constant *α*_*i*_ [/m] as:

1 $$ E_{i} = \alpha_{i} \;V = \alpha_{i} V_{0} \sin 2\pi ft $$

2 $$ v_{i} = \mu\,E_{i} $$

where *V*_*0*_, *f*, *v*_*i*_, *μ* are the amplitude of the applied voltage [V], frequency [Hz], velocity of the target ion (at position *i*) [m/s], and mobility of the ion [m^2^/Vs], respectively. The amount of ion *Q*_*i*_ [C/m^2^] passing through the unit area at position *i* from time *τ*_*1*_ [s] to *τ*_*2*_ [s] is:

3 $$ \begin{aligned} Q_{i} = & \int\limits_{{\tau_{1} }}^{{\tau_{2} }} {qv_{i} n_{0} N_{A} dt} \\ = & \frac{{q\mu \alpha_{i} V_{0} n_{0} N_{A} }}{2\pi f}\left( {\cos 2\pi f\tau_{1} - \cos 2\pi f\tau_{2} } \right) \\ \end{aligned} $$

where *q*, *n*_*0*_, *N*_*A*_ are the elementary charge [C], ion concentration at infinite distance from the electrode (bulk of the suspension) [mol/m^3^], and Avogadro constant [/mol], respectively. From Eq. (3), defining the outer side of the membrane as “out” and the inner side as “in”, the voltage between the membrane *ΔV* [V] is expressed as:

4 $$ \begin{aligned} \Delta V = & \frac{{Q_{out} - Q_{in} }}{C} = \frac{{q\mu (\alpha_{out} - \alpha_{in} )V_{0} n_{0} N_{A} }}{2\pi fC}\left( {\cos 2\pi f\tau_{1} - \cos 2\pi f\tau_{2} } \right) \\ = & \frac{{q\mu (r - 1)\alpha_{in} V_{0} n_{0} N_{A} }}{2\pi fC}\left( {\cos 2\pi f\tau_{1} - \cos 2\pi f\tau_{2} } \right) \\ \end{aligned} $$

where *C* is the capacitance of the membrane [F/m^2^]. We defined *r* as $r = \frac{{\alpha_{out} }}{{\alpha_{in} }}$. In the vicinity of the cylindrical electrode, the intensity of the electric field and its change depending on location are large, so we considered Eq. (4) within the range of *r* > 1.

Potassium ions can pass through biological membranes more easily than other ions due to the activities of membrane proteins such as transporters^19^. We calculate the value of *r* assuming that *ΔV* is 75 mV at the membrane near the electrode in Eq. (4). Assuming that *q* = 1.6 × 10^–19^ C, *N*_*A*_ = 6.0 × 10^23^/mol, *V*_*0*_ = 1.2 V, *f* = 14 Hz, the potassium ion concentration *n*_*0*_ = 30 × 10^–3^ M (in the suspension), *α*_*in*_ = 100/m (reciprocal of the distance between electrodes), *τ*_*1*_ = 0 s, and *τ*_*2*_ = *T*/2 s (*T* = 1/*f,* the accumulation of potassium ions over half cycle), and using values of *μ* = 7.6 × 10^–8^ m^2^/Vs for potassium ion mobility and *C* = 1.0 × 10^–2^ F/m^2^ for the capacitance of the membrane^17^, we found that *r* was approximately 1.0013. In other words, if the difference in intensity of the electric field between the inside and outside surface of the membrane is 0.13%, a voltage of about 75 mV is generated.

The current–voltage characteristics of potassium ion channels and transporters present in the membrane of the yeast have been reported based on the patch clamp method, and application of a negative voltage of several tens of mV to the inside of the cell relative to the outside causes potassium ions to flow into the cell in a manner depending on the voltage^3,4^.

**Figure 3:**
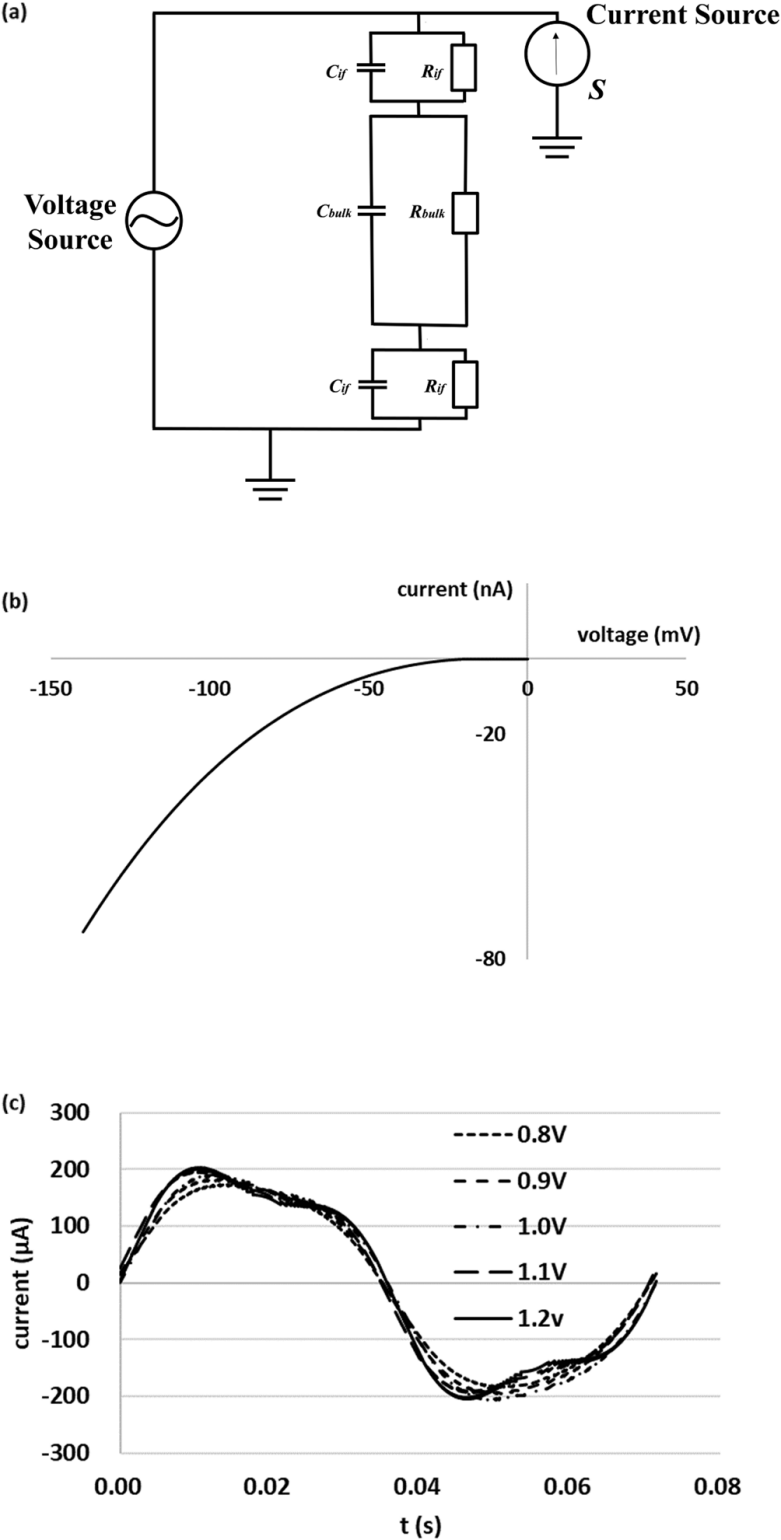
(**a**) Equivalent circuit used in the simulation. (**b**) Current–voltage characteristics of the potassium ion transporter used in the simulation. (**c**) Results of the equivalent circuit simulation at applied voltages of 0.8, 0.9, 1.0, 1.1, and 1.2 V at 14 Hz using the current–voltage characteristics of the potassium ion transporter.

To explore the cause of the difference in the unique distortion pattern of the waveform of living yeast depending on the applied voltage, we simulated waveforms assuming an influx of potassium ions. Figure 3a shows the equivalent circuit. In Fig. 3a, *R*_*if*_ and *C*_*if*_ are the resistance and capacitance at the electrode-suspension interface, respectively. *R*_*bulk*_ and *C*_*bulk*_ are the resistance and capacitance in the bulk of the suspension, respectively. In addition to these linear elements, the current source *S*, which is nonlinear with respect to the voltage across the membrane, is set as the influx of ions into the cell via ion channels and transporters present in the yeast cell membrane. We suggest that the current generated by the transfer of ions through the membrane near both the positive and negative electrodes flows inward, as the intensity of the electric field is high near the electrode and the positions of the electrodes are symmetrical. Assuming that the values of *R*_*if*_, *C*_*if*_, *R*_*bulk*_, *C*_*bulk*_ were constant when the frequency was set to 11, 14, 17, or 19 Hz at 0.5 V, we found that *R*_*if*_ = 0.6 kΩ, *C*_*if*_ = 2.6 μF, *R*_*bulk*_ = 14 kΩ, and *C*_*bulk*_ = 3.7 μF. Figure 3b shows the current–voltage characteristics of current source *S* in the simulation. The value of the horizontal axis represents the voltage applied to the inside of the cell relative to the outside, and we considered the characteristics of only the negative voltage range (inward-flowing current) here. These characteristics are shown for one yeast cell, and its form is based on a previous study^4^. Because the experimental conditions in that study differ from those in this study, such as strains and growth conditions, the values were set between those used in references^3,4^. In Fig. 3b, we used 75 mV as the applied voltage at the membrane when a voltage of 1.2 V was applied to the suspension. For other applied voltages, we used values proportional to the amplitude of the applied voltage. Further, the phase angle of the current source was in phase with that of the flowing current.

Simulation of the flowing current in the circuit was performed for each applied voltage at 14 Hz using the LT-SPICE model. Figure 3c shows the results of that simulation. Assuming that the cell concentration was 4 × 10^8^ cells/mL, the yeast cells were spherical with a diameter of 8 μm, and the number of cells was constant near the electrode, we multiplied 1.3 × 10^5^, representing the number of yeast cells, by the value of the current shown in Fig. 3b. In reality, the membrane of each yeast cell is under unique electric field intensity conditions; however, to perform the simulation, we assumed that a difference existed in the intensity of the electric field between the inside and outside membrane surface, which was uniform for all yeast cells. In the results of the simulation with varying applied voltages, the generation of distortion depending on the applied voltage aligned with the waveform in the experimental results. However, the change in current, corresponding to that in the applied voltage, was smaller than the corresponding change in the experimental results. Moreover, smaller currents were obtained than in the experimental results.

**Figure 4:**
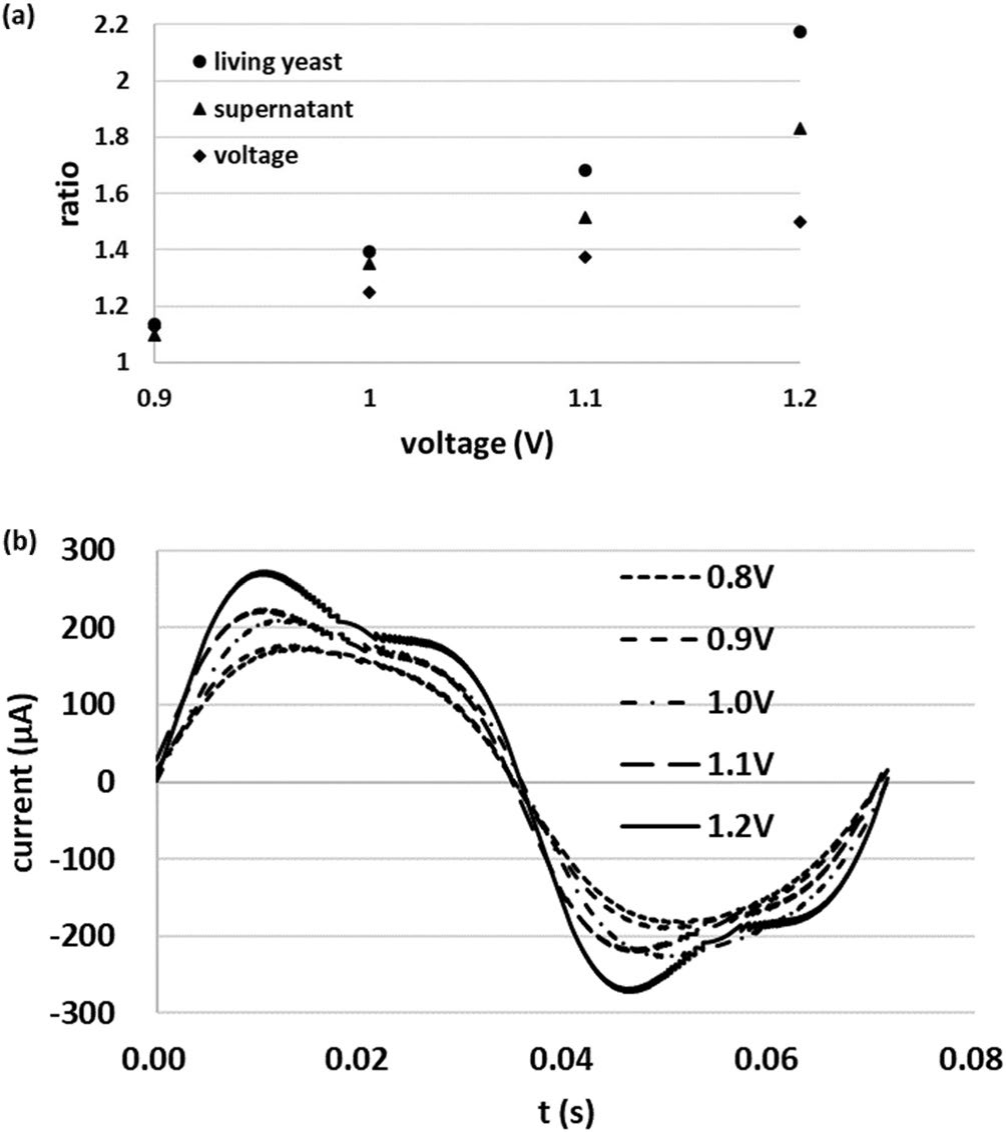
(**a**) Ratio of the maximum current value of the living yeast suspension and its supernatant at each voltage to that at 0.8 V, and ratio of each applied voltage to 0.8 V. (**b**) Results after modification.

The current waveform of the medium from living yeast (supernatant obtained through centrifugation of the living yeast suspension) has no distortion (data not shown), as observed for dead yeast. Figure 4a shows the ratio of the maximum experimental value of living yeast suspension, its supernatant at each applied voltage (0.9 V, 1.0 V, 1.1 V, and 1.2 V) to that of 0.8 V and each applied voltage to 0.8 V, respectively. We found that the ratio of the supernatant was larger than that of each applied voltage. In the suspension, the values were greater than in the supernatant. To focus on the effect of the living yeast on the generation of nonlinearity, we added the difference based on the difference between the ratio of the supernatant and that of each applied voltage, shown in Fig. 4a, to the simulation results shown in Fig. 3c (Fig. 4b). Greater similarity was found between this result and the experimental result for the dependence on applied voltage. However, the current remained smaller than those obtained in the experimental results. This difference may have arisen because we considered only negative voltage applied to the membrane near the electrode, while in reality a positive voltage may be applied to the membrane. The assumption that a voltage of 75 mV was applied to the membrane near the electrode may have driven this difference. The similarity in the distortion pattern between the results depending on applied voltage and the experimental results suggests the validity of this model.

## Conclusions

We showed the current passing through yeast suspensions with the application of AC voltage, and found that the nonlinearity of the waveform depended on the applied voltage and frequency. Furthermore, we suggested a model for the nonlinearity and validated it by performing a simulation using an electric circuit. We showed that the influx of potassium ions into the cell could generate the nonlinearity. It is important to investigate the effect on cell growth and/or metabolism of the influx into (and outflow from) cells of ions upon application of a voltage as well as the application as a biosensor such as monitoring the state of cells by measuring the current. Accumulation of experimental data related to yeast cells and their membranes and further assessment of the similarity between simulations and experimental results will provide insight into the current–voltage characteristics of the cell membrane in vivo.
