# Author Correction: Generation of nonlinearity in the electrical response of yeast suspensions

**DOI:** 10.1038/s41598-022-08614-1

**Published:** 2022-03-15

**Authors:** K. Tamura, M. Muraji, K. Tanaka, T. Shirafuji

**Affiliations:** Graduate School of Engineering, Osaka City University, Sumiyoshi-ku, Sugimoto, 558-8585 Japan

Correction to: *Scientific Reports* 10.1038/s41598-022-07308-y, published online 04 March 2022

The original version of this Article contained a repeated error in the x-axis labels of Figure 2 (a), (b), (d), (e), (f), (g), where the data points 0, 0.02, 0.04, 0.06 and 0.08 were incorrectly given.

The original Figure 2 and accompanying legend appear below.

**Figure 2:**
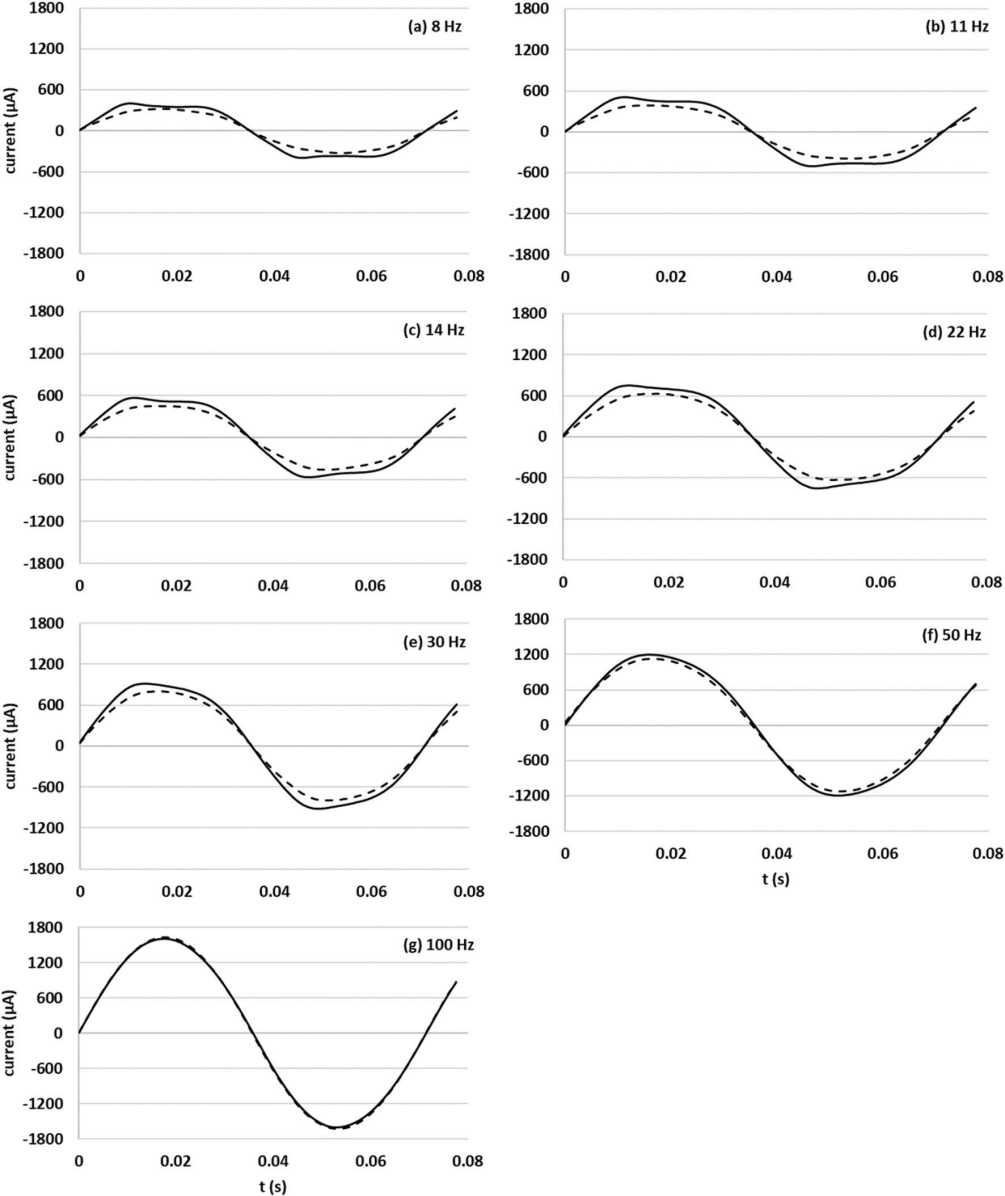
(**a**)–(**g**) Current waveforms of living and dead yeast 90 s after measurement was initiated at frequencies of 8, 11, 14, 22, 30, 50, and 100 Hz at 1.2 V.

The original Article has been corrected.

